# Children and young people’s perceptions of energy drinks: A qualitative study

**DOI:** 10.1371/journal.pone.0188668

**Published:** 2017-11-30

**Authors:** Shelina Visram, Stephen J. Crossley, Mandy Cheetham, Amelia Lake

**Affiliations:** 1 School of Medicine, Pharmacy and Health, Durham University Queen’s Campus, Stockton-on-Tees, United Kingdom; 2 Fuse (UKCRC Centre for Translational Research in Public Health), Newcastle University, Newcastle-upon-Tyne, United Kingdom; 3 School of Health and Social Care, Teesside University, Middlesbrough, United Kingdom; McMaster University, CANADA

## Abstract

**Background:**

Consumption of soft drinks is declining in many countries, yet energy drink sales continue to increase, particularly amongst young consumers. Little is currently known about the drivers behind these trends. Energy drinks are high in sugar and caffeine, and evidence indicates that regular or heavy use by under 18s is likely to be detrimental to health. This study aimed to explore children and young people’s attitudes and perceptions in relation to energy drinks in a UK context.

**Methods:**

Eight focus groups were conducted with pupils aged 10–11 years (n = 20) and 13–14 years (n = 17) from four schools in northern England. A sub-sample also took part in a mapping exercise to generate further insights. Data were analysed using the constant comparative approach.

**Results:**

Energy drinks were reportedly consumed in a variety of public and private places, generally linked to social activities, sports and computer gaming (particularly amongst boys). Participants demonstrated strong brand awareness and preferences that were linked to taste and perceived value for money. The relatively low price of energy drinks and their widespread availability were identified as key factors, along with gendered branding and marketing. Some participants demonstrated a critical approach to manufacturers’ claims and many were keen to become better informed, often through school- or peer-based interventions. Other potential interventions included age restrictions, voluntary schemes involving retailers and improved labelling.

**Conclusions:**

The lack of a single dominant factor in participants’ consumption choices suggests that there is unlikely to be a ‘silver bullet’ in attempting to address this issue. However, the findings provide support for policy-level interventions that seek to change the behaviours of manufacturers and retailers as well as consumers, and actively involve children and young people where possible.

## Introduction

Consumption of sugar-sweetened beverages (SSBs) by children and young people (C&YP) is a growing public health concern. This is due to the long-term implications associated with excessive sugar intake, which include dental erosion, weight gain and the development of obesity and type 2 diabetes [1]. Evidence from the UK indicates that consumption of SSBs is highest amongst those aged 13 to 18 years, both in relative and absolute terms (548 KJ or 41% of energy intake), in comparison with adults and younger children [2]. Consumption of SSBs increased throughout the 20^th^ century in high income countries such as the UK, USA and Australia, but is now declining due to consumer concerns over added sugars [3]. Energy drinks (EDs), which are characterised by their high caffeine content (>150mg per litre), represent one category of SSB that has not followed this trend. Sales of EDs in the UK increased by 185% between 2006 and 2015, equating to 672 million litres consumed in 2015 and a total market value of over £2 billion [4]. The global EDs market, worth $50 billion, is projected to grow at an annual rate of 3.5% until at least 2020 [5]. Several low- and no-sugar ED varieties have been launched but their caffeine content remains high. Furthermore, high-sugar varieties still remain popular and represent one of the main sectors driving growth for the soft drinks industry as a whole [3].

Evidence suggests that regular or heavy ED use is likely to be detrimental to C&YP in the short- and long-term [6, 7]. Correlational studies have identified links with common health complaints such as headaches, stomach aches and sleeping problems, which increase in prevalence with greater ED use [8, 9]. There is also strong evidence that youth ED consumption clusters with other health-damaging behaviours, including binge drinking, smoking, illicit drug use, screen time and poor dietary behaviours [10–13]. There have been calls to restrict the sale of EDs in recognition that childhood and adolescence are periods of rapid growth and brain development, when adequate sleep and good nutrition are especially important [6]. However, these drinks are increasingly popular amongst young consumers. A 2011 survey conducted across 16 European Union (EU) countries found that prevalence of ED consumption was highest amongst 10 to 18-year-olds (68%, compared with 30% of adults and 18% of younger children) [14]. Furthermore, this age group in the UK consumed more EDs on average than their counterparts in other EU countries (3.1 litres per month, compared with 2 litres). Recent analysis of international sales data indicated that sales of EDs rose sharply between 2010 and 2015, and that the UK had the second highest rate of ED sales per head globally [15]. Little is currently known about the drivers behind these trends.

Quantitative, survey-based research suggests that C&YP tend not to differentiate between energy and isotonic/sports drinks, and that they consume both for reasons that include taste, to quench their thirst, and to improve sports performance [16–18]. Many of these studies have been conducted in North America or the Middle East, highlighting a need for further research to explore potential differences in motivations, experiences and expectations amongst C&YP from different geographies. The aforementioned European survey found that C&YP consume EDs at home, during sport and exercise, and at parties [14]. However, less is known about where and how they obtain these drinks. A separate body of literature exists in relation to consumption of EDs by young adults, often examining the experiences of university or college students [19–21]. Much of this literature focuses on co-consumption with alcohol (AmED) and generally finds that this behaviour is associated with a range of negative outcomes, which in many cases are worse than those associated with ED or alcohol consumption alone [22–24]. AmED consumption amongst young adults appears to be motivated by hedonistic tendencies, as well as being linked to societal norms of masculinity [25, 26].

Qualitative research on this topic is limited. Four published studies–all conducted in Australia or New Zealand–have examined ED consumption by C&YP [27–30]. Of these, one also involved over 18s, one explored use of EDs pre-mixed with alcohol, and another examined use of various nutritional supplements (of which EDs were one category). Further in-depth research is needed to understand the motivations of C&YP in the UK (without the emphasis on co-consumption with alcohol) given that, on average, they consume EDs more often and in higher quantities than those in many other countries [14]. The aims of this study were to explore the attitudes and perceptions of C&YP in relation to EDs, and to seek their views on possible intervention options. The study had the following research questions:

1. Why, when and how do some C&YP choose to consume EDs, and why do others choose to abstain?
2. Where, from whom and at what cost do C&YP obtain EDs?
3. What are the implications of these findings for policy and practice?

## Material and methods

### Procedure

A data-driven qualitative study design was chosen to meet the study aims and research questions. The study involved C&YP and researchers working in partnership to combine knowledge and action on EDs, thereby drawing on the principles of co-production and community-based participatory research (CBPR) [31]. The design was informed by guidelines for ethical research with C&YP [32]. The study received ethical approval from the School of Medicine, Pharmacy and Health Research Ethics Sub-Committee at Durham University (ref. ESC2/2014/08).

### Participants

A pragmatic approach was taken in drawing the study population from localities suggested by the project advisory group, which comprised local health, education and community engagement practitioners as well as the research team. Eight schools in County Durham, northern England, were approached and four (two primary and two secondary) agreed to take part in the study. All were situated in areas characterised by relatively high levels of socio-economic deprivation. Students from Year 6 (aged 10–11 years) and Year 9 (aged 13–14 years) were recruited to the study via their class teachers or form tutors. These age groups were chosen based on team members’ experience of conducting similar studies with school-age C&YP, coupled with advice from the project advisory group. Year 6 is a key transition year between primary and secondary school in the UK, whereas Year 9 is arguably the final year before the pressures and workload of significant exams begin (and when fewer students are likely to have begun experimenting with alcohol). Preliminary discussions with school staff and a local parent group also indicated that ED consumption was perceived to be a growing problem amongst both age groups.

Teachers were encouraged either to randomly select potential participants or, if they felt this was not appropriate, to select diverse groups of students (i.e. with different levels of educational attainment). Up to five boys and five girls from each class were chosen and provided with age-appropriate study information packs to consider before deciding whether or not to take part. All participants gave their written informed consent to take part in the study. Parents/carers were provided with separate information and invited to opt-out of the study by reply. A non-response was taken as indication of assent for their child to take part, in an effort to reduce potential biases associated with an opt-in approach.

### Data collection

The primary mode of data collection involved semi-structured focus groups, conducted (by SJC and MC) using a topic guide developed to address the research questions (Box 1). The benefits of this method included the explicit use of group interaction to produce additional data and insights [33, 34]. Potential risks included participants seeking to impress their peers or the researchers by either exaggerating or understating their level of ED use; therefore, they were asked to talk generally about ED consumption amongst their classmates rather than their own experiences. Single-sex groups were conducted, based on guidance from the literature [35] and team members’ experience of research involving similar age groups. The format was kept flexible and informal to enhance comfort and foster openness. The focus groups began with a sorting exercise (using empty cans and bottles of EDs, isotonic/sports drinks and soft drinks) to generate discussion on the participants’ understandings of the differences between these drinks. The groups lasted between 20 and 45 minutes and were held on school premises, during school hours, without school staff present. All discussions were audio-recorded with participants’ consent.

**Box 1. Focus group topic guide**

- Sorting exercise and general discussion on the differences between energy drinks, sports/isotonic drinks and soft drinks
- Consumption patterns: when and where EDs are consumed; reported prevalence amongst their classmates; gender and age differences
- Accessiblity
- Cost
- Marketing and branding
- Reasons for use or non-use
- Effects: benefits and potential risks or harms
- Policy and practice within their school
- Possible interventions

**Fig 1:**
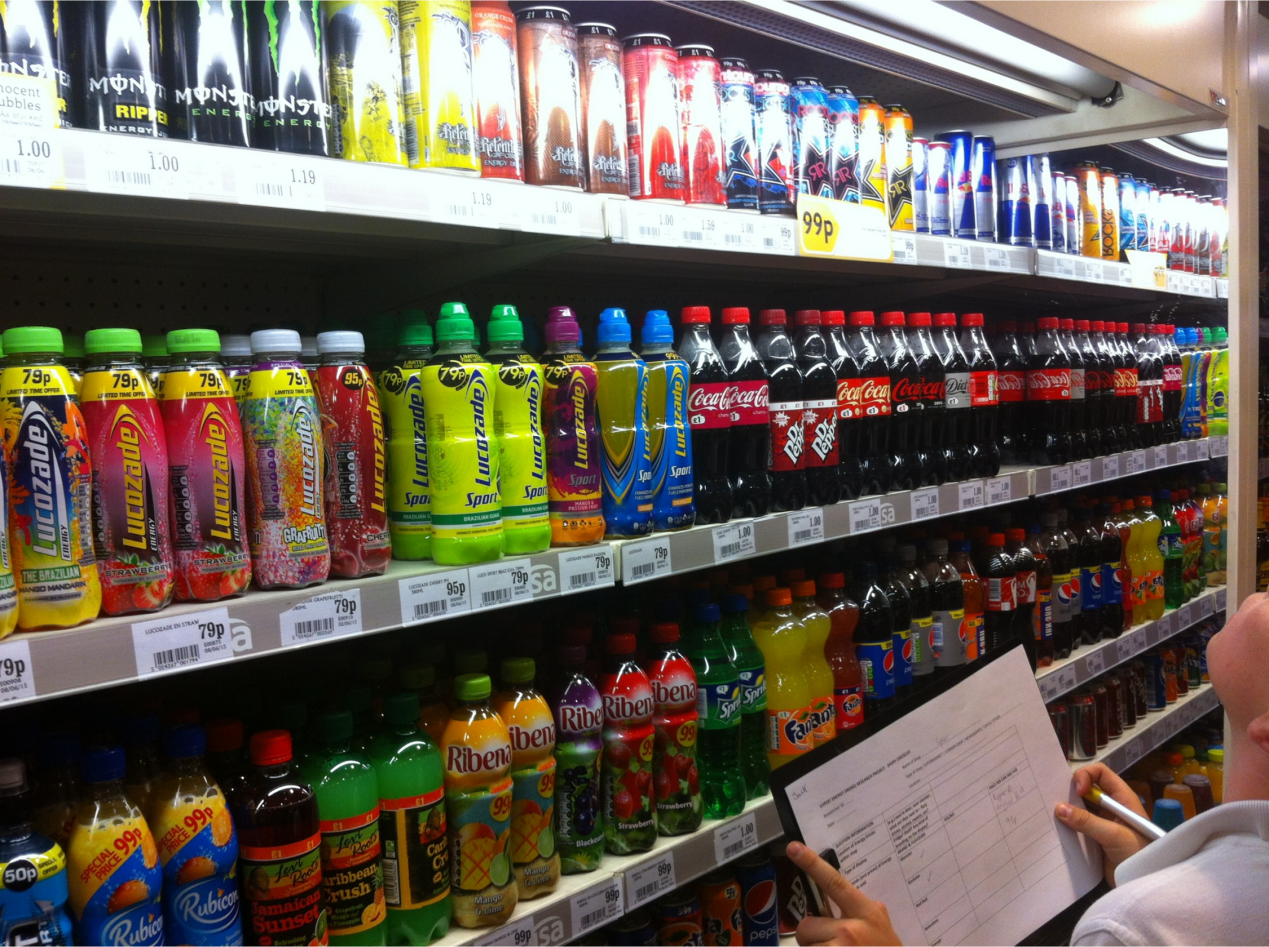
Photograph taken during participatory mapping exercise.

The study also involved a mapping exercise conducted by a sub-sample of participants (2–4 from each school). Three of the four schools permitted their students to take part in this exercise, which involved spending up to one hour walking around the local area (accompanied by at least one researcher and a member of school staff) to identify outlets where EDs could be purchased. The purpose was to involve C&YP in the process of generating data, enabling them to gain basic research skills and allowing the research team to gain greater insight into the availability and accessibility of EDs. A template was designed to record the location, brands, prices and sizes of the drinks available, as well as the price and availability of other beverages (isotonic/sports drinks, soft drinks and water), based on earlier food environment work [36]. Photographs were taken, inside or outside shops, where appropriate. See Fig 1 for an example.

### Data analysis

The focus group recordings were transcribed verbatim, anonymised and analysed using the constant comparative approach, which meant that themes were generated inductively using a data-driven approach as opposed to applying an *a priori* coding framework informed by previous research or driven by existing theory [37]. NVivo qualitative analysis software v.10 was used to systematically organise and index materials. Initial analysis of four focus groups was undertaken (SJC and MC) to identify emerging themes from the data; primary codes were identified and then discussed with the wider research team. Themes were checked and further codes identified following subsequent analysis. Preliminary findings were discussed with the advisory group, to obtain feedback on the researchers’ interpretations and to generate further discussion on the implications for policy and practice.

Marketing rapidly emerged as a key theme and therefore the marketing mix was applied towards the end of the analytical process as a framework to better understand drivers for consuming EDs [38]. Often referred to as ‘the four Ps’–product, price, place and promotion–the marketing mix is central to the planning and implementation of integrated marketing strategies. This framework has been adapted from the commercial sector in planning social marketing programmes that seek to achieve behavioural goals for social good [39, 40]. In the present study, three additional Ps–peers, parents/carers and policy–emerged through the analytical process, and the data were subsequently categorised according to this conceptual framework (Table 1).

**Table 1:** The enhanced marketing mix.

	Ps	Details/sub-themes
**The 4 Ps of commercial marketing**	**Product**	• Knowledge of ED contents and their effects (risks and benefits) • Taste as primary motivator • Preference for particular brands and packaging types
	**Price**	• EDs perceived as cheaper than other beverages • C&YP motivated to seek value for money • Price increases as a possible intervention
	**Place**	• Widespread availability • Consumed in a variety of settings: public spaces, at home, on the way to and from school
	**Promotion**	• Varied marketing tactics of ED companies • Links to sport and computer gaming
**Additional Ps that emerged during the study**	**Peers**	• Social norms • Gendered practices • Potential for peer-led interventions
	**Parents and carers**	• Facilitating or limiting access to EDs • Limited influence on C&YP’s consumption choices
	**Policy**	• Realistic expectations about possible interventions: - Sales restrictions - Voluntary retail schemes - Educational interventions - School food policies

## Results

A total of 37 C&YP consented to participate in the study (19 males and 18 females; 20 aged 10–11 years and 17 aged 13–14 years). Findings relating to each element of our enhanced marketing mix are reported in turn below, illustrated using direct quotations from the participants.

### Product

Participants in both age groups appeared to have a good level of knowledge about certain aspects of EDs, including brand names, key ingredients and some of the potential risks associated with consumption. However, there were areas where confusion and uncertainty were evident. The high caffeine content was often highlighted as a defining characteristic of EDs, but there appeared to be less understanding of what constituted high levels of caffeine or sugar:

*There’s more sugar in them [points to ED] than what’s in them [points to sports drink]*, *because there’s about 10 spoons of sugar I think in them*. (Girl, 13–14 years)

*Some younger kids they read [the label] but say they don’t know what… 4*.*8 sugars mean*. *They don’t know what it means–like*, *is that a lot or is it not a lot?* (Girl, 10–11 years)

It was suggested that information about sugar and caffeine content could be presented on EDs in ways that are easier for C&YP to understand; for example, in spoonfuls of sugar. Other suggestions included using plain English on packaging (instead of unfamiliar terms like insomnia), featuring a ‘Think before you drink’ sign, or making the text about potential health risks more visible:

Girl 1: *I think they should just make it bigger writing*.

Girl 2: *Make it more in your face… Like eye-catching*. (Exchange between two girls, 13–14 years)

The sweet and distinctive flavour of EDs reportedly made them particularly appealing to C&YP. Taste was identified as one of the main influences in the decision to consume these products, although others also spoke about using them when they felt tired and needed a physical or mental ‘boost’ (often linked to consuming EDs in order to stay up late, creating a cyclical effect):

*Everybody only drinks them for their taste*. *They don’t really want them for the hyper; they just want them for the taste*. (Boy, 13–14 years)

*If you want to be woken up you drink the big ones*. (Girl, 13–14 years)

A preference for particular brands and packaging sizes was highlighted as an important factor in determining the quantities consumed. Most participants who consumed EDs reported a preference for cheaper varieties in smaller cans as opposed to some of the better-known premium brands. For others, the larger cans or bottles were preferred because “*you get more for your money*".

### Price

The relatively low cost of some ED varieties, particularly in relation to other beverages, was identified as a key factor in C&YP’s decisions to purchase these drinks:

*Because they’re only cheap and plenty of people get money*. *So instead of looking for one of them [points to sports drink]*, *that’s 95p at that shop down there*, *they look at the energy drinks*, *that’s only 35p*. (Girl, 10–11 years)

*I think it’s because like a normal can of Coke is like 70p*, *and [EDs] are like 35p*. (Girl, 13–14 years)

The mapping exercise confirmed that ‘own brand’ EDs available from local convenience stores were amongst the cheapest drinks on sale (*“cheaper than water and pop”*). It also revealed that these drinks were often included in multi-purchase offers, where buying more than one product results in a discount. Some C&YP reported pooling their money and sharing EDs with friends to take advantage of special offers or the proportionately lower price for large cans or bottles:

*You know them little cans*, *like the Emerge?* *You can get*, *like*, *four for £1*. (Girl, 13–14 years)

*We get a big bottle of Emerge and share it*. (Boy, 13–14 years)

A minority of participants suggested that increasing the cost of EDs could help to reduce consumption, although there was some disagreement around the most appropriate price level:

Girl 1: *They could step up the prices without them knowing*. *Because one day it could be 45p and then the other day it can be £4 or £5 or something*, *and it could like stop them…*

Girl 2: *They should be about £1*.*50*. *Like*, *kids our ages doesn’t get about that much*.

Girl 3: *They should stay the price they are*. (Exchange between three girls, 10–11 years)

### Place

EDs appeared to be easily accessible to C&YP, with one participant reporting that, “*every shop apart from the pet shop and the furniture shop sells energy drinks”*. The mapping exercise confirmed their widespread accessibility and availability, with most convenience stores offering numerous brands, flavours and packaging sizes. They tended to be positioned near or opposite the entrance and were often given greater shelf or fridge space than other drinks. Some participants suggested that the positioning of EDs within stores could be changed in order to restrict access by C&YP:

*I think they should go in their own aisle where children are not allowed*, *like cigarettes*, *and they should all be together*. *And we’re not allowed… we shouldn’t go in that aisle*. (Girl, 10–11 years)

C&YP reported consuming EDs in a wide variety of places, including public spaces such as on the street, in parks or at leisure facilities, including football pitches, BMX tracks and skate parks. Much of the discussion focused on the journey to and from school. However, some participants described consuming EDs at home, often linked to computer gaming and, less often, to sleepovers:

Girl 1: *Most kids drink them on the morning on the way to school… Or on the night walking home*.

Girl 2: *Or when you’re out with your mates on the night*. (Exchange between two girls, 13–14 years)

*Sometimes it’s sleepover day when your mates come round*. *So you go to the shop*, *you get loads of*, *you get energy drinks and you go in your house and you just play on your X-Box and stuff like that*. (Boy, 10–11 years)

### Promotion

In addition to seeing EDs for sale in local shops, participants identified a range of different media through which they were targeted with promotional messages about ED products. These included: the internet (in the form of ‘pop-ups’ or banners at the side of webpages); television (including ED consumption or product placement during popular shows); computer games; bus-stop adverts; supermarket promotions; and sponsorship of sports or other events:

*If you’re playing on your tablet or something and you’re playing a game*, *an advert pops up for like Relentless*. (Girl, 10–11 years)

*It sponsors like big BMX games and stuff like that… Yeah*, *like extreme sports generally and stuff*, *it like sponsors all of them and that*. (Boy, 13–14 years)

As a consequence, participants in both age groups displayed a strong awareness of the major ED brands, and many were familiar with particular advertising slogans and strategies. They perceived that the branding and packaging of these drinks made them attractive to C&YP. Some were identified as targeting boys in particular, through the brand names, packaging colours and sizes, and the association with extreme sports:

*I think that the drinks are a little bit sexist because they have all these boyish kind of patterns*, *but you never see any girly ones*. (Girl, 10–11 years)

C&YP reported that certain EDs have links with particular computer games, for example, via codes under the ring-pull which could be used to boost the stamina of characters. All of the games mentioned carried age restrictions and were marketed as being suitable for players 18 years and over, yet many of the boys in our focus groups were familiar with them:

*There’s a game called Dead Island where you sometimes get them to fill your health up*. (Boy, 10–11 years)

*[In the game Saint’s Row] you own this energy drink company and you start selling them to gangs and everything*. (Boy, 10–11 years)

It was suggested that ED companies have an important role to play in marketing these products in age-appropriate ways, although there was little discussion about stronger forms of regulation or restrictions on advertising. Some participants were sceptical of the promotional claims made by ED companies in adverts and on packaging. Many were aware that these tactics were used to encourage people to buy products, thereby generating additional profit for manufacturers:

*Once I was playing on this game and the Monster advert popped up*, *and it said ‘Sugar-free*, *fat-free*, *caffeine-free*, *and good for you’*. *And I was like*, *“No"*. (Girl, 10–11 years)

*They put [promotional codes] on so that Monster can make more money*. (Boy, 13–14 years)

### Peers

There was a general perception amongst participants in both age groups that ED usage was widespread within their peer groups. Some made a distinction between regular consumers and those who had tried EDs once or not at all:

*About everyone’s tried one*, *but I don’t think they keep on trying it*. *About five or four people haven’t tried them or had a drink of them*. (Boy, 10–11 years)

Consumption appeared to be highly gendered, in that boys were perceived as being more likely to drink EDs, and in greater quantities, than girls. This was often associated with boys reportedly being more active and more likely to take part in sports-related activities:

Girl 1: *Boys are most likely to drink them… Yeah*, *because they’re playing football and things*.

Girl 2: *They like sporty things in the school*.

Girl 3: *They run out of energy so they think that when they drink them they get more*, *but actually they lose energy*. (Exchange between three girls, 10–11 years)

Boys’ use of EDs was often described as linked to wanting to appear tough or attractive to girls, whereas girls expressed a preference for ‘expensive-looking’ brands in smaller cans which they associated with being sophisticated:

*If you have a girlfriend or something*, *like*, *boys like to drink them then*, *because they think… Sometimes the boys think that they look proper rock-hard when they have them in front of girls and stuff*. *And then when they go out*, *you buy like three*. (Boy, 10–11 years)

A number of participants believed that their peers drank EDs in order to enhance their image or identity. These influences were generally described as affecting other C&YP, rather than being reported as important factors in their own consumption decisions. However, there was some evidence of boys and girls choosing to consume EDs as part of desire to ‘fit in’:

*Say your friend*, *he has something that looks really*, *really nice–an energy drink*, *like a Monster–but you only have a bottle of water*. *You’re tempted to get that because it looks cooler and what your friends have you want*, *so you don’t want to be left behind*. (Girl, 10–11 years)

C&YP also reported positive instances where friendships groups had collectively decided to abstain or cut back on ED usage, as illustrated by the quote below. This highlighted the potential for peer-led interventions, for example, training C&YP as champions to provide information and advice on EDs to their peers. The involvement of C&YP in designing and developing interventions was a theme which ran through the focus group discussions.

Boy 1: *Every morning we used to get them and we used to down them*.

Boy 2: *Then we stopped because then we knew what the effects were*.

Boy 1: *So we just got the 35p ones*. *We didn’t get the big ones*. (Exchange between two boys, 10–11 years)

### Parents and carers

Parents, carers and other adults played a role in C&YP’s decisions to consume EDs, either by facilitating or limiting their access or by modelling this behaviour. Participants gave examples of family members who drank EDs and were ambivalent about C&YP also drinking them, while others were more firmly ‘against’ these products. Some reported that parents had provided EDs to rehydrate after sports activities or as a treat for doing chores:

*Sometimes [dad] says to me*, *“Go down the shop*, *go to [name of shop] and get us*, *I don’t know*, *some washing up liquid”*. *And he says*, *“There’s a can of Red Bull in there with your name on it”*. (Boy, 10–11 years)

Others mentioned relatives refusing to allow them to consume EDs, generally because of concerns about possible negative health and behaviour-related effects. Examples were given of other adult role models, such as sports coaches, influencing this behaviour:

*[Coach says] “Don’t drink them before football*, *just bring some water”*. *[…] The coach just cares for you and he wants to look out for you*. *And he doesn’t want your heart full of junk*. (Boy, 10–11 years)

Most participants reported purchasing EDs themselves, often using lunch money or ‘pocket money’. Given that EDs tended to be consumed in a range of settings outside of the home, family members were not always aware of this behaviour. As a consequence, parents and carers were not generally identified by C&YP as important actors in discussions about possible intervention options.

*Some parents don’t allow people to drink energy drinks*, *so they just drink them when they’re out*. (Girl, 13–14 years)

### Policy

Many participants suggested that there should be age restrictions on EDs, similar to those in place for cigarettes and alcohol. However, there was little agreement on what would be an appropriate age limit, with suggestions ranging from eight to 18 years (linked to the age of the participants, i.e. generally suggesting that consumption should be limited for children younger than themselves). Furthermore, most believed that this type of intervention could be easily overcome by asking older friends, siblings or parents to purchase EDs on their behalf. Regardless, participants generally acknowledged that an age limit would help to send a clear message that these products are not recommended for C&YP:

*[The packaging] doesn’t exactly say*, *‘Don’t give it to someone under age 16’ […] They should be banned for children… Like under 16*, *15*. (Girl, 10–11 years)

*I think you maybe should make a certain age for certain ones*, *such as Monster maybe*. *No*, *Relentless– 16*. *Maybe Monster*. (Boy, 13–14 years)

In the absence of legislation regarding sale of EDs to C&YP, several participants suggested voluntary schemes involving local retailers. They identified local shops or, more commonly, individual employees who voluntarily restricted sales to C&YP:

*There’s this old woman that works in [name of shop] and she doesn’t sell*, *she reads all the cans before she sells you any drink*. *But then it depends who’s serving you*. (Girl, 10–11 years)

This approach was believed by some to be unworkable because of the challenges of implementation with large national retailers and the importance of ED-related income to smaller retailers. Instead, school-based interventions were suggested as a potential way of raising awareness of the potential effects of EDs and prompting voluntary behaviour changes. Some C&YP were confused about precisely how their health could be affected, offering opportunities to discuss EDs during assemblies, biology lessons, or personal, health, social and economic (PHSE) education:

*Yeah*, *because if you have a lesson on them*, *you might get to understand them more and you know that they’re bad for you*. *And so you just shouldn’t drink them*. (Girl, 10–11 years)

Existing school food policies appeared to inhibit ED consumption for the majority of C&YP during school hours, particularly in primary schools. However, examples were given of pupils finding ways around these rules and also of school staff lacking knowledge of EDs, highlighting the importance of education for adults as well as C&YP:

*I don’t think that some teachers understand what the energy drinks are*. *Like*, *sometimes we bring [sports drinks] into school and the teachers say they’re energy drinks when they’re not*, *like the isotonics [sports drinks]*. *And sometimes we’ll be drinking energy drinks but they won’t notice*. (Boy, 13–14 years)

## Discussion

### Summary of key findings

This qualitative study explored attitudes and perceptions of C&YP in relation to EDs, with a view to informing potential interventions. The findings are reported here around themes derived from an enhanced version of the traditional marketing mix (or 4 Ps)–product, price, place, promotion, peers, parents/carers and policy. Participants in both the younger and older groups demonstrated strong brand awareness and preferences that were linked to taste and perceived value for money. The relatively low price of many EDs and their widespread availability were identified as key factors in C&YP’s purchasing decisions. The accessibility of these drinks was confirmed through the mapping exercise, which highlighted the wide range of products available, the low cost of some brands, and the promotional offers available in many shops. Parents, carers and other significant adults had a role to play in facilitating or limiting access to EDs, or by helping to normalise their use. Gendered branding and marketing also emerged as influential factors. Some participants demonstrated a critical approach to the marketing tactics and nutritional claims made by ED manufacturers, and were keen to become better informed.

C&YP reported consuming EDs in a variety of public and private places, generally linked to social activities, sports and computer gaming (particularly amongst boys). There was some evidence of C&YP choosing EDs as part of a desire to ‘fit in’ or ‘look tough’. But, on the whole, our discussions appeared to suggest autonomous, rational decision-making; for example, friendship groups ‘clubbing together’ to make purchases more affordable or choosing to cut back or abstain from EDs as a group. This complexity in C&YP’s ED use has implications for the design and implementation of future interventions, which participants advised should involve C&YP as far as possible. Suggestions included age restrictions, voluntary schemes involving retailers, and improved labelling and marketing of EDs. School food polices and restrictions imposed by individual shopkeepers appeared to inhibit ED consumption for many C&YP, but no single policy or practice intervention was felt to represent a ‘silver bullet’ to address this complex issue.

### Comparison with existing literature

The study complements findings from previous research on use of EDs by C&YP, but also highlights complexities that may be more pronounced in countries with high levels of ED consumption like the UK. Several studies have identified taste as a key motivating factor, with the perceived stimulant properties and performance enhancing effects of EDs representing secondary motivators [17, 27–29, 41–43]. Advertising and brand loyalty were also identified as important influences [44]. Similarities between the marketing tactics of major carbonated drinks manufacturers and the tobacco companies of previous decades have already been highlighted [45, 46]. There are also obvious parallels with alcohol marketing [47]. The youth-oriented marketing and branding activities of ED companies would benefit from further scrutiny, particularly in terms of links to sport, gaming, sexuality and gender, and wider risk-taking behaviours. In a focus group study involving three age groups (16–21, 22–28, 29–35 years), industry marketing was perceived as targeting specific drinks at males or females, using sexualised imagery and humour [27]. Brand loyalty emerged as a common theme across all groups, but the youngest age group appeared to be most conscious of the social image they were portraying in their choices. In the present study, both age groups and genders demonstrated awareness of major brands and popular advertising slogans. To our knowledge, this is the first study to explore in-depth the views of children as young as 10 years in relation to EDs.

Previous studies highlight a perception of EDs as costly; in one case being described as ‘twice as expensive as traditional soft drinks’ [48]. An Australian focus group study involving students aged 11–18 years found that some reported using EDs as soft drink substitutes, but only when they could afford them [30]. A recent blog identified that the cheapest EDs on sale in Australian convenience stores start from $3.50, which equates to around £2.00 [49]. This contrasts with the findings of the present study, where C&YP perceived EDs to be cheaper than many other beverages and the mapping exercise identified that they could be purchased for as little as 35p. Price and value for money were clearly of high importance to these age groups. Similar results in terms of C&YP being price conscious have been found in relation to food purchasing, where participants reported ‘shopping around’ to get what they considered to be value for money [36]. A key difference between the findings of the present study and previous research relates to awareness of potential negative consequences associated with ED consumption. Qualitative studies highlight limited understanding of possible risks, particularly amongst younger age groups [27, 29, 30]. Participants in a 2003 study talked enthusiastically about the perceived beneficial effects on their bodies and their sports performance, but made no mention of any harmful effects [30]. In the present study, school food policies and educational interventions appeared to have increased awareness of ED contents and potential risks associated with their consumption. However, this did not appear to prevent participants from purchasing a cheap, readily available drink that was perceived to taste good and was heavily marketed towards them, suggesting a need to move beyond individual health education approaches to addressing wider structural influences on this behaviour.

The social meanings and social context of ED consumption emerged as important influences on participants’ choices about where, when and what they consumed. Research on C&YP’s relationships with food brands shows that consumption choices may be used to support the image they choose to project and to express affinity with particular social or peer groups [50]. Peer influences are often assumed to be negative, but research shows that C&YP can also influence one another’s health-related behaviours in positive ways [51]. Rather than reducing their choices to ‘peer pressure’ or a simple desire to look ‘cool’, participants in this study articulated a far more nuanced picture in discussing their perceptions, preferences and practices in relation to EDs. They supported policy-level interventions and had realistic expectations about what might be feasible in attempting to reduce ED consumption. This research contributes to our understanding of the complex influences on health-related decision making by C&YP, and highlights the relevance of critical perspectives informed by C&YP’s views gathered in qualitative research. Further research involving other stakeholders (i.e. teachers, parents, manufacturers and retailers) and conducted in different contexts would help to shed further light on this type of complex behaviour. Comparative studies in particular are needed to answer the question of why ED consumption in the UK is so high.

### Strengths and limitations

This study had several limitations. Participants were located in one geographical area within northern England and the findings may not be generalizable to other areas, particularly given the lack of area-level prevalence data and dearth of studies on ED use conducted in a UK context. Furthermore, data were collected in and around schools and provide insights from participants at a particular time and place. This research does not tell us about patterns of ED consumption amongst C&YP more widely. While the design included efforts to reduce bias, there remains the possibility of selection bias in the convenience sample of schools who agreed to take part in the study and also in the recruitment of students by teachers. Two parents opted out of allowing their children to participate in the study; we have no means of ascertaining the reasons for their decision. None of the C&YP opted out.

There existed a risk of social acceptability bias, in that participants may have felt they were going to be judged for reporting high ED use or, conversely, may have exaggerated their intake and associated risky behaviours. Participants were asked to talk about EDs in general, or the perceived behaviours of their friends and classmates, in an effort to reduce this risk. Although many also shared their own experiences, we did not gather data specifically on levels of ED consumption. Single gender focus groups were chosen to maximise opportunities for participation and informal feedback indicated that girls in particular liked this approach. However, it may have informed the data in ways we did not anticipate. The combination of practical sorting exercises and discussion-based activities appeared to work well, with the empty cans and bottles often acting as prompts for further discussions on branding and content. Including a participatory mapping exercise also enriched the study findings. C&YP engaged enthusiastically with the research process and feedback indicated that they found the discussions enjoyable. Overall, we believe that our approach was successful in generating valuable insights on awareness and consumption of EDs by C&YP.

## Conclusions

This is one of the first studies to explore C&YP’s perceptions of EDs in a UK context, where prevalence of ED consumption is particularly high and the cost of many ED products is relatively low. Taste, price, promotion, ease of access and peer influences were all identified as key factors in C&YP’s consumption choices. The lack of a single dominant factor suggests that there is unlikely to be a ‘silver bullet’ in attempting to address this issue. However, the importance of value for money to C&YP and the strong influence of the marketing activities of ED companies should not be underestimated. These findings provide support for policy-level interventions that seek to change the behaviours of manufacturers and retailers as well as consumers, and actively involve C&YP as far as possible.

## Abbreviations

AmED: Alcohol mixed with energy drinks
C&YP: Children and young people
ED: Energy drink
SSB: Sugar-sweetened beverage
